# Age-Dependent Association Between Insomnia and Body Mass Index Among Young Women: Evidence from a Cross-Sectional Study

**DOI:** 10.3390/jcm14248904

**Published:** 2025-12-16

**Authors:** Anna Merklinger-Gruchała, Anna Goździalska, Agnieszka Bień, Joanna Grzesik-Gąsior

**Affiliations:** 1 Faculty of Health Sciences, Medical College, Andrzej Frycz Modrzewski Krakow University, Herlinga-Grudzińskiego 1, 30-705 Kraków, Poland; amerklinger-gruchala@uafm.edu.pl (A.M.-G.); agozdzialska@uafm.edu.pl (A.G.); 2 Chair of Obstetrics Development, Faculty of Health Sciences, Medical University of Lublin, 20-081 Lublin, Poland; agnieszka.bien@umlub.edu.pl; 3 State University of Applied Sciences in Krosno, 38-400 Krosno, Poland

**Keywords:** insomnia, body mass index, women, age factors, shift work

## Abstract

**Background/Objectives:** Sleep disturbances, particularly insomnia, are increasingly recognized as important determinants of health. Previous studies have shown bidirectional associations between sleep quality and obesity. Limited evidence exists on the modifying role of age in the relationship between insomnia symptoms and body mass index (BMI) among women. This study aimed to evaluate the association between insomnia symptoms and BMI in women, with a specific focus on the potential effect of age. **Methods:** A cross-sectional study was conducted among 72 female nursing students aged 21–48 years. Data were collected via an online questionnaire including demographic, occupational, and anthropometric variables, as well as the Athens Insomnia Scale (AIS) to assess insomnia symptoms. BMI was calculated based on self-reported weight and height. Logistic and multiple linear regression models were used to evaluate the relationship between AIS scores and BMI and age. **Results:** Overall, insomnia severity was not directly associated with BMI in the full sample (*p* = 0.55). However, a significant interaction between insomnia symptoms and age was observed (*p* = 0.02). Among women aged ≥37 years, higher AIS scores were positively associated with BMI (β = 0.59; *p* = 0.06), whereas this association was absent in younger participants. **Conclusions:** Insomnia symptoms showed a trend toward a positive association with higher BMI, primarily among older women, suggesting an age-dependent relationship between sleep disturbances and body weight. Screening for sleep problems and promoting sleep hygiene could serve as simple, low-cost preventive strategies for maintaining metabolic health in women.

## 1. Introduction

Sleep is a physiological process that plays a fundamental role in the regulation of metabolic, hormonal, and cardiovascular functions in the human body. Sleep disorders are now recognized as common health complaints affecting large segments of the population. They may involve disturbances in sleep quality, timing, or duration, including difficulties with sleep initiation, nocturnal awakenings, early morning awakenings, or the inability to return to sleep. Insomnia is the most prevalent sleep disorder, accounting for approximately 90% of all sleep-related complaints. Its reported prevalence varies widely, from 4.4% to 48%, largely due to the subjective nature of the condition and difficulties in establishing consistent diagnostic criteria. According to the International Classification of Diseases (ICD-10), the key diagnostic feature of insomnia is a difficulty in initiating or maintaining sleep that leads to impaired daytime functioning and reduced quality of life. Symptoms must occur at least three times per week for a minimum duration of one month, and the condition cannot be attributed to another medical or psychiatric disorder [1].

Women are more likely than men to experience insomnia across various age groups [2]. This higher prevalence among women may be attributed to a combination of psychosocial and biological factors, including caregiving responsibilities, an increased risk of depression, and hormonal fluctuations throughout the lifespan [3].

Sleep disturbances, including insomnia, can contribute to unfavorable changes in body composition through several physiological mechanisms [4,5]. Sleep restriction leads to a decrease in leptin levels and an increase in ghrelin secretion—hormones that regulate appetite—resulting in increased hunger and caloric intake. Consequently, insufficient sleep promotes weight gain and higher body mass index (BMI). Moreover, poor sleep quality may negatively affect glucose metabolism and insulin sensitivity. Conversely, obesity itself constitutes a significant risk factor for sleep disturbances, including insomnia and obstructive sleep apnea, suggesting a bidirectional relationship between sleep and body weight regulation [4,5,6]. Circadian rhythm disruption is common among shift workers and has been shown to profoundly affect metabolic homeostasis. Misalignment between internal circadian timing and external behavioral cycles disrupts glucose tolerance, alters cortisol and melatonin secretion, and increases adiposity even in the absence of caloric excess. Such chronobiological misalignment may therefore represent an important underlying mechanism linking sleep disorders with metabolic dysregulation and obesity risk [7].

This relationship is further influenced by age-related changes. As individuals age, sleep architecture undergoes characteristic modifications: sleep latency increases, nighttime awakenings become more frequent, and overall sleep efficiency declines [8]. At the same time, metabolic and hormonal changes occur, which predispose individuals both to weight gain and sleep disturbances, creating a vicious cycle linking aging, obesity, and deteriorating sleep quality [9].

Given these findings, there is a clear need to evaluate the interrelationship between insomnia symptoms, BMI, and age in specific populations, particularly among individuals exposed to chronic stress and irregular work schedules. To date, few studies have examined this association in young, professionally active women, a group in which sleep problems may coexist with the early onset of metabolic alterations. Understanding this relationship could provide valuable insights into the pathophysiological mechanisms linking sleep disturbances with metabolic risk and serve as a basis for the development of preventive strategies aimed at improving sleep quality and weight control.

The present study aimed to assess the association between insomnia symptoms, body mass index, and age among women working in shift-based employment settings.

## 2. Materials and Methods

### 2.1. Study Design and Participants

A cross-sectional study was conducted to examine the relationship between insomnia symptoms, body mass index (BMI), and age among women performing shift-based or irregular work. The study utilized a self-administered online questionnaire distributed via the Computer-Assisted Web Interviewing (CAWI) method. Data collection was carried out between 20 October 2023 and 31 January 2024 using a digital survey tool (Google Forms). The questionnaire included demographic and lifestyle questions (age, type of employment, duration of professional experience, and work schedule—shift work, single day shift, single night shift, or no employment) as well as anthropometric measures (self-reported body weight and height) used to calculate BMI. Insomnia symptoms were assessed using the Athens Insomnia Scale (AIS), a validated Polish version based on ICD-10 criteria [10], which evaluates difficulty initiating and maintaining sleep, early morning awakening, and daytime functioning over the preceding three months. The AIS demonstrates high sensitivity (93%) and specificity (85%), and permission to use the validated Polish version was obtained from the copyright holders.

The study population consisted of nurses aged 21–48 years in Kraków, Poland. This group was selected because of their exposure to irregular schedules and high prevalence of sleep disturbances, making them an informative cohort for exploring the relationship between sleep quality, age, and metabolic health. Inclusion criteria were as follows: biological sex female, active menstruation, and voluntary participation. Exclusion criteria included the following: pregnancy, lactation, menopause, diagnosed endocrine disorders (e.g., polycystic ovary syndrome or thyroid dysfunction), psychiatric or eating disorders, and the use of sleep medications or hormonal therapy.

The dataset used in this analysis originated from the same broader survey on women’s health and sleep patterns described previously [11]. However, the present study focuses specifically on the association between insomnia, BMI, and age.

### 2.2. Ethical Considerations

The research protocol was approved by the University Bioethics Committee (No. KBKA/55/O/2023; 19 October 2023). Each participant provided informed consent electronically before participation and was informed of the study’s objectives and voluntary nature. Respondents could withdraw at any time. No personally identifiable or sensitive data were collected in this anonymous study.

### 2.3. Statistical Analysis

Statistical analyses were performed using Statistica version 14.1.0.4 (TIBCO Software Inc., Palo Alto, CA, USA) and jamovi Computer Software (Version 2.6, The jamovi project 2025, Sydney, Australia). Descriptive statistics were used to summarize the characteristics of the study population. Continuous variables were expressed as mean  ±  SD and median and quartiles (Q1, Q3) and compared using a Mann–Whitney U Test. The categorical variables were expressed as frequency (n, %). The χ^2^ test or Fisher’s exact test were used to compare groups. The normality of distribution was assessed using the Shapiro–Wilk test.

Simple and multiple logistic regression was used to examine the association between AIS total score and odds of overweight (BMI > 25), with the interaction term (AIS × age) included to examine whether the relationship between insomnia and BMI differed across age groups. Potential confounders, including age, self-perceived PMS symptoms, shift work, and age at menarche, were included in adjusted models. ROC analysis was performed to determine an optimal age cut-off for women’s age, determined by Youden’s method. Multiple logistic regression analyses were repeated using categorized age based on this cut-off. Additionally, models were repeated using BMI as a continuous outcome after Box–Cox transformation. Model fit was evaluated using McFadden’s R^2^, Nagelkerke R^2^, and overall chi-square tests. Due to the Box–Cox transformation, standardized regression coefficients (β) were reported instead of unstandardized coefficients, as the latter took extremely small values (on the order of 10^−5^) and could not be meaningfully interpreted in BMI units. Standardized β coefficients indicate the number of standard deviations by which the dependent variable changes for each one standard deviation increase in the predictor, while holding all other variables constant. Statistical significance was set at *p* < 0.05. Confidence intervals (95% CI) were calculated for all regression coefficients. A post hoc power analysis was conducted using G*Power (version 3.1., Heinrich-Heine-Universität, Düsseldorf, Germany).

## 3. Results

The study included 72 women, aged 21–48 years, with body weights ranging from 48.0 to 102.0 kg. The majority of women participating in the study worked as nurses, n = 67 (91.8%). Among the participants, 26.0% (*n* = 19) were classified as overweight (BMI > 25). In the comparative analysis between women with normal weight and those who were overweight, several demographic and anthropometric differences were observed. Women who were overweight were significantly older (*p* < 0.01), had higher body weight (*p* < 0.01), and shorter height, although the difference in height did not reach statistical significance (*p* = 0.14). The age at menarche did not differ significantly between groups (*p* = 0.89). Similarly, the severity of insomnia symptoms, as assessed by the Athens Insomnia Scale (AIS), did not differ significantly between normal-weight and overweight participants (*p* = 0.58). Overweight status showed no association with the prevalence of symptoms suggestive of premenstrual syndrome (*p* = 0.46). The normal weight and overweight groups did not differ significantly across work schedule (shift work vs. single shift/not working; *p* = 1.00) or monthly workload (hours) (*p* = 0.63). However, work seniority was significantly associated with the occurrence of overweight (*p* < 0.01): people with the longest work experience (>5 years) were significantly more likely to have a BMI ≥ 25 compared to the other groups. The participants characteristics according to overweight status was presented in Table 1.

**Figure 1:**
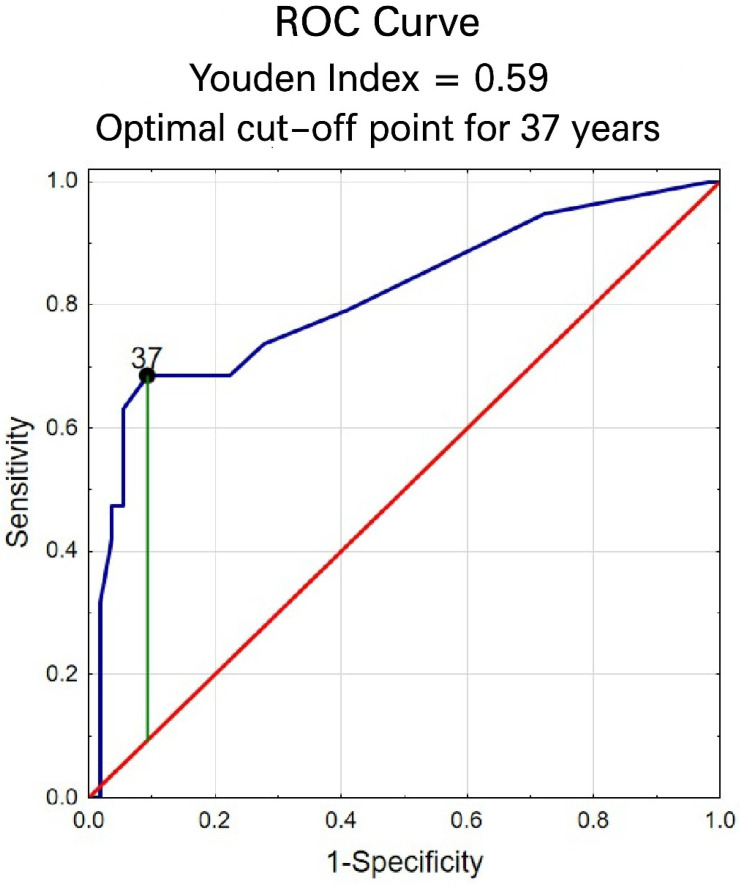
ROC curve for age (blue line) in predicting overweight (BMI > 25). The red diagonal line indicates no discrimination. The green line represents the maximum Youden’s index used to determine the optimal cut-off value (37 years; AUC = 0.81, 95% CI: 0.68–0.94).

A Receiver Operating Characteristic (ROC) analysis was performed to identify an optimal cut-off point for women’s age, using overweight (BMI > 25) as the outcome variable. The cut-off value was determined using Youden’s index and is presented in Figure 1. The optimal age threshold was 37 years, which predicted overweight status with a sensitivity of 68% and a very high specificity of 91%. The area under the ROC curve (AUC) was 0.81 (95% CI: 0.68–0.94), indicating good discriminative ability of the model. Since an age of ≥37 years correctly identified more than two-thirds of women with overweight, this cut-off was adopted for subsequent analyses and used as a categorical variable in further regression models.

Among women aged ≥37 years, a consistent trend was observed indicating slightly poorer sleep quality and next-day functioning, although the differences compared with younger participants were small (Table 2). Older women (≥37 years) demonstrated similar or slightly better sleep initiation efficiency (*p* = 0.03) and fewer early awakenings (*p* = 0.32). However, they scored higher on several Athens Insomnia Scale (AIS) items, including total sleep duration, overall sleep quality, next-day well-being, mental and physical functioning after awakening, and daytime sleepiness. The group of women with overweight (BMI > 25) also presented with higher total AIS scores compared to those with normal BMI; however, this difference was not statistically significant (*p* = 0.90).

**Figure 2:**
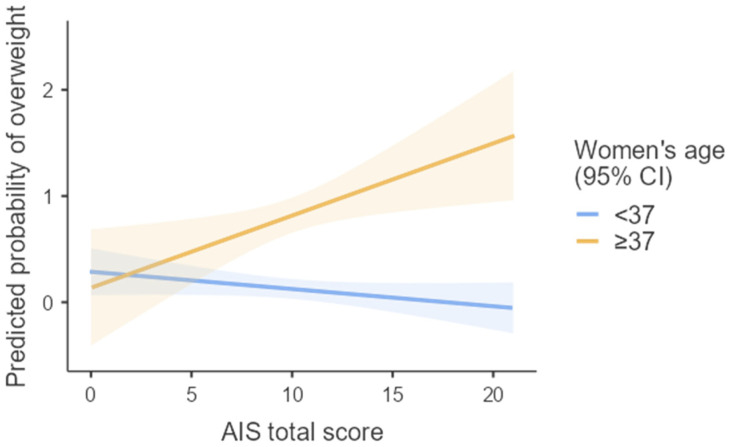
Probability of overweight by AIS total score, stratified by maternal age (<37 vs. ≥37 years). Shaded areas: 95% CIs. Age moderated the insomnia–overweight association (interaction *p* = 0.016), with a logistic regression model adjusted for PMS-like symptoms, shift work, and age at menarche (McFadden’s R^2^ = 0.42, Nagelkerke R^2^ = 0.56, overall model test *p* < 0.001).

No significant association was found between insomnia severity (AIS) and overweight status, either before adjustment (OR = 0.95, 95% CI: 0.84–1.08) or after adjustment for potential confounders, including age, premenstrual syndrome symptoms, work schedule, and age at menarche (OR = 0.95, 95% CI: 0.79–1.14). However, a statistically significant interaction was observed between AIS total score and age (*p* < 0.001). Based on the ROC analysis, which identified 37 years as the optimal age cut-off, we repeated the multiple logistic regression analysis using age as a categorical variable (<37 vs. ≥37 years). In this model, the interaction term remained significant (*p* = 0.02, Figure 2), indicating that the relationship between insomnia and overweight was modified by age. Among women aged ≥37 years, the association between AIS total score and overweight reached borderline statistical significance (OR = 1.54, 95% CI: 0.99–2.40), whereas in the younger group (<37 years), the relationship was non-significant (OR = 0.83, 95% CI: 0.65–1.06).

We repeated the adjusted regression models, treating BMI as a continuous outcome variable after Box–Cox transformation, and obtained similar results. Among younger women (<37 years), higher insomnia severity (AIS total score) was associated with higher BMI (*β* = 0.59, 95% CI: −0.04 to 1.21); however, this effect did not reach statistical significance (*p* = 0.06). The interaction term (age group × AIS total score) remained statistically significant (*p* = 0.032), indicating that the relationship between insomnia symptoms and BMI differed by age. Among older women (≥37 years), a positive trend was observed: greater insomnia severity was associated with higher BMI (*β* = 0.59, 95% CI: −0.03 to 1.21), a result that approached statistical significance (*p* = 0.06). In contrast, among younger women (<37 years), the association was negative but not statistically significant (β = −0.13, 95% CI: −0.36 to 1.10, *p* = 0.26).

In summary, age significantly moderated the association between insomnia severity and BMI, suggesting that the relationship between sleep disturbances and body weight may differ between younger and older women.

## 4. Discussion

Sleep deprivation is associated not only with subjective discomfort but also with an increased risk of multiple somatic conditions, including metabolic disorders, hypertension, ischemic heart disease, and depression [7,12,13]. Insomnia, while highly prevalent, rarely occurs in isolation. It often coexists with other sleep disturbances that include short sleep duration, sleep fragmentation, circadian rhythm misalignment, and sleep-disordered breathing. These disturbances are highly prevalent across all age groups and are increasingly recognized as key contributors to physical and mental health risks [14]. By framing insomnia within this broader context, the current findings underscore the public health importance of addressing sleep health not only as an individual clinical concern but as a modifiable population-level determinant of metabolic outcomes [12].

The present study provides new evidence that the association between insomnia symptoms and body mass index in women may vary with age, after adjustment for self-perceived PMS symptoms, age at menarche, and shift work. Further standardization of work seniority did not affect these results. While no overall relationship was observed between insomnia severity and BMI in the entire sample, the analysis indicated a positive trend between insomnia and BMI among women aged ≥37 years, but not among younger participants. A stronger association between insomnia and BMI in women over 37 years of age may reflect cumulative effects of age-related physiological and hormonal changes. In this life stage, subtle declines in estrogen and progesterone, alterations in melatonin secretion, and increased vulnerability to stress-related HPA axis activation may jointly exacerbate the metabolic impact of poor sleep [15]. Additionally, lifestyle factors such as reduced physical activity and increased professional and caregiving demands might further strengthen this relationship in midlife women [16].

Although insomnia is not a physiological component of the aging process, this finding may indicate that with increasing age, individuals could become more susceptible to the potential metabolic consequences of sleep deprivation. The obtained results are consistent with previous population-based studies and meta-analyses. Chan et al. demonstrated that individuals suffering from insomnia were more likely to be overweight or obese, with this association being stronger among women [6]. Similarly, Figorilli et al. confirmed a bidirectional relationship between sleep disturbances and obesity—not only does sleep loss promote weight gain, but excess body weight may also exacerbate insomnia symptoms, particularly through mechanisms involving obstructive sleep apnea and chronic low-grade inflammation [5].

However, the current analysis extends this evidence by showing that age may act as a moderating factor, amplifying the effect of insomnia on body weight. Several studies have indicated that middle-aged women, particularly those experiencing hormonal changes and chronic stress, are more likely to report both sleep difficulties and increased adiposity [17,18].

Several plausible mechanisms may underlie this age-dependent association. In midlife, women experience hormonal shifts, including declines in estrogen and progesterone, which can affect both sleep regulation and fat distribution. These hormonal changes may lead to reduced sleep efficiency, increased awakenings, and impaired thermoregulation, which in turn could affect appetite regulation and glucose metabolism [19,20,21].

Chronic insomnia has been hypothesized to contribute to neuroendocrine and metabolic disturbances that promote excessive weight gain. Research has shown that sleep restriction alters hypothalamic signaling pathways responsible for appetite control and increases the preference for carbohydrate-rich, high-energy foods. In addition, chronic sleep deprivation may impair glucose metabolism and promote elevated cortisol secretion, which in turn facilitates the accumulation of visceral fat [21,22]. Together, these findings are consistent with the hypothesis of a bidirectional relationship between sleep disturbances and metabolic dysregulation, whereby insomnia contributes to weight gain, while excess adiposity and chronic inflammation further deteriorate sleep quality [23,24]. Age appears to amplify this interaction, as hormonal resilience and circadian adaptability decline over time [22].

Not only insomnia symptoms, but also short sleep duration (less than 7 h per night) was found to be associated with a higher mean body mass index and an increased risk of obesity. Sleep restriction leads to elevated ghrelin levels and reduced leptin secretion, resulting in enhanced appetite and greater caloric intake [7,25]. Nikpour et al. reported that among women aged 25–45 years, this relationship may be further amplified by irregular circadian rhythms, shift work, and psychosocial stress [26]. In younger individuals, shorter sleep duration may be partially compensated by higher energy expenditure and more efficient metabolism, whereas in adults over 25 years of age, the negative metabolic and hormonal consequences of sleep disruption gradually accumulate [9]. Among women, advancing age is also associated with a decline in estrogen and progesterone levels, which can affect thermoregulation and circadian rhythm stability. Consequently, older women may be more susceptible to sleep disturbances, and their metabolic consequences become more pronounced. In contrast, younger women may benefit from protective factors such as favorable hormonal profiles, higher physical activity, and more adaptive physiological mechanisms [20,27,28].

Behavioral factors, such as decreased physical activity and increased caregiving responsibilities, may also interact with these biological mechanisms to amplify the observed association in older participants. Notably, reduced muscular strength and the presence of musculoskeletal pain, both associated with insomnia, may contribute to sedentary behavior and additional metabolic risk in this population [29]. Environmental stressors, such as household disorganization, have also been associated with disrupted sleep in parents, suggesting that psychosocial factors may further compound metabolic vulnerabilities in women managing multiple roles [30].

The results of this study highlight the clinical importance of incorporating sleep assessment into metabolic risk screening, particularly among women over 35 years of age. In this age group, many women experience occupational and psychosocial stressors, including shift work, which may contribute to both sleep disturbances and metabolic dysregulation. Interventions focusing on sleep hygiene, stress management, and lifestyle modification could play a valuable role in reducing the risk of overweight and obesity in clinical practice. Moreover, incorporating a brief screening question on sleep quality into routine preventive examinations within primary health care may represent a cost-effective long-term preventive strategy for promoting metabolic and overall health in women.

Furthermore, future studies should explore the potential association between body mass index (BMI) and care complexity. Care complexity is a dynamic and multidimensional construct shaped not only by patients’ medical conditions but also by their functional status, psychosocial needs, and overall symptom burden. Recent evidence suggests that sleep disturbances, such as insomnia, short sleep duration, and circadian misalignment, frequently co-occur with other symptoms in individuals experiencing high psychosocial or clinical vulnerability and may meaningfully contribute to increased care demands. For example, Cesare et al. (2024) demonstrated that higher nursing care complexity, measured through the number of nursing diagnoses and actions, was associated with an increased risk of intra-hospital and ICU transfers in pediatric patients, even after accounting for chronic conditions [31].

In a subsequent study, the same research group found that care complexity did not consistently correlate with the number of chronic conditions, suggesting that other factors, including multidimensional symptom clusters such as sleep-related problems, may independently contribute to nursing workload and resource needs [32]. From this perspective, integrating sleep-related variables and metabolic indicators such as BMI into future assessments of care complexity could yield valuable interdisciplinary insights and inform more personalized healthcare strategies.

This study, however, has certain limitations. First, its cross-sectional design precludes the determination of a causal relationship between insomnia and overweight. The data on body weight and height were self-reported, which may have slightly underestimated actual BMI values. Furthermore, the study did not include information on caffeine intake, physical activity, diet, or use of hormonal contraceptives—factors that may influence both sleep quality and body weight. In addition, the homogeneity of the sample, which consisted exclusively of female nursing students from a single city and spanned a relatively narrow age range, may limit external validity. This was an intentional design choice to focus on women of reproductive age and reduce hormonal heterogeneity, allowing for a clearer examination of age-dependent patterns in the relationship between insomnia symptoms and BMI. Importantly, nursing students and nurses are frequently used in sleep research because they represent a group at elevated risk of sleep disturbances due to irregular schedules, high stress, and demanding work environments. This makes them an informative population for studying sleep and metabolic outcomes associations. Nevertheless, we agree that future studies including more diverse occupational, geographic, and age groups, particularly peri- and postmenopausal women, are needed to confirm and extend these findings to broader populations.

The sample size (N = 72) was relatively small. A post hoc power analysis was conducted for the incremental effect of the AIS × age interaction in the multiple linear regression model. Introducing the interaction term increased the explained variance by ΔR^2^ = 0.098, corresponding to an effect size of f^2^ = 0.098. With a total sample size of N = 72 and six predictors included in the model, the achieved statistical power for detecting the interaction was 0.74, as estimated using G*Power (F test, R^2^ increase). This value falls below the conventional 0.80 benchmark, indicating that the interaction effect should be interpreted cautiously and ideally confirmed in a larger sample. Nonetheless, the observed interaction highlights an interesting direction for future research.

Previous studies examining the relationship between insomnia and obesity have focused primarily on general or clinical populations, while adult women, who often combine academic or professional responsibilities with high psychological stress and irregular circadian rhythms, have received relatively little attention. Assessing this relationship within such a group may provide a better understanding of the early mechanisms underlying metabolic disturbances and help identify modifiable risk factors that could inform preventive strategies and targeted interventions.

## 5. Conclusions

The severity of insomnia symptoms showed a tendency toward a positive association with higher BMI, particularly among women over 37 years of age, suggesting, but not yet confirming, an age-dependent relationship between sleep disturbances and body weight. These preliminary findings point to the potential importance of considering sleep health as part of metabolic risk assessment for women of reproductive age. As insomnia symptoms appear to influence body weight more strongly in older age, targeted sleep interventions and lifestyle counseling may play a preventive role in mitigating the risk of overweight and obesity.

Considering the high prevalence of insomnia among women exposed to stress and shift work, early screening and education on sleep hygiene could contribute to improved metabolic and overall health outcomes. Promoting good sleep hygiene and monitoring sleep quality could represent simple yet effective strategies to support women’s long-term metabolic well-being.

## Figures and Tables

**Table 1:** Characteristics of participants according to overweight status.

Characteristic	Overweight (BMI > 25)		*p*-Value
	No (n = 54)	Yes (n = 19)	
Age (years), Me (Q1–Q3)	23.0 (22.0–25.0)	40.0 (24.0–45.0)	<0.01
Age category, n (%)			<0.01
<37	49 (89.1%)	6 (10.9%)	
≥37	5 (27.8%)	13 (72.2%)	
Menarcheal age (years), Me (Q1–Q3)	13.0 (12.0–14.0)	13.0 (12.0–14.0)	0.89
Body mass (kg), Me (Q1–Q3)	60.0 (57.0–65.0)	75.0 (68.0–83.0)	<0.01
Height (cm), Me (Q1–Q3)	166.0 (164.0–169.0)	164.0 (160.0–167.0)	0.14
Symptoms suggesting PMS, n (%)			0.46
No	15 (68.2%)	7 (31.8%)	
Yes	39 (76.5%)	12 (23.5%)	
Shift work, n (%)			1.00
Single shift/not working	13 (76.5%)	4 (23.5%)	
Shift work	41 (73.2%)	15 (26.8%)	
Work seniority, n (%)			<0.01
No work experience	4 (80.0%)	1 (20.0%)	
1 month–1 year	22 (100.0%)	0 (0.0%)	
1–5 years	21 (80.8%)	5 (19.2%)	
>5 years	7 (35.0%)	13 (65.0%)	
Monthly workload (hours), n (%)			
0 h (not working)	5 (83.3%)	1 (16.7%)	
1–85 h (≤0.5 FTE *)	1 (100.0%)	0 (0.0%)	
86–175 h (≤1 FTE *)	40 (75.5%)	13 (24.5%)	
176–200 h	8 (61.5%)	5 (38.5%)	
AIS (total score), Me (Q1–Q3)	10.5 (7.0–14.0)	11.0 (7.0–13.0)	0.63

* FTE—full-time equivalent.

**Table 2:** Athens Insomnia Scale (AIS) item scores by age group (<37 vs. ≥37).

	Age Group		
AIS Item	<37 (N = 55)	≥37 (N = 18)	*p*-Value
Falling asleep after going to bed, mean (SD)/median (Q1–Q3)	1.3 (0.9)/1.0 (1.0–2.0)	0.8 (0.5)/1.0 (1.0–1.0)	**0.03**
Night-time awakenings, mean (SD)/median (Q1–Q3)	1.0 (0.9)/1.0 (0.0–2.0)	0.9 (0.4)/1.0 (1.0–1.0)	0.88
Early morning awakenings, mean (SD)/median (Q1–Q3)	0.7 (0.9)/0.0 (0.0–1.0)	0.4 (0.8)/0.0 (0.0–1.0)	0.32
Total sleep duration, mean (SD)/median (Q1–Q3)	1.4 (0.8)/2.0 (1.0–2.0)	1.7 (0.5)/2.0 (1.0–2.0)	0.18
Overall sleep quality, mean (SD)/median (Q1–Q3)	1.3 (0.8)/1.0 (1.0–2.0)	1.4 (0.7)/2.0 (1.0–2.0)	0.54
Well-being the next day, mean (SD)/median (Q1–Q3)	1.3 (0.9)/1.0 (1.0–2.0)	1.6 (0.7)/2.0 (1.0–2.0)	0.26
Physical and mental functioning the next day, mean (SD)/median (Q1–Q3)	1.1 (0.8)/1.0 (1.0–2.0)	1.4 (0.7)/2.0 (1.0–2.0)	0.10
Daytime sleepiness, mean (SD)/median (Q1–Q3)	1.7 (0.7)/2.0 (1.0–2.0)	1.8 (0.5)/2.0 (2.0–2.0)	0.44
AIS (total score)	9.9 (4.5)/10.0 (7.0–14.0)	10.2 (3.2)/11.0 (8.0–13.0)	0.90

## Data Availability

The raw data supporting the conclusions of this article will be made available by the authors on request.

## Abbreviations

The following abbreviations are used in this manuscript:

AIS	Athens Insomnia Scale
BMI	Body Mass Index
CAWI	Computer-Assisted Web Interview (web-based survey)
PMS	Premenstrual Syndrome
